# Mechanical Properties of Bulk Metallic Glasses Additively Manufactured by Laser Powder Bed Fusion: A Review

**DOI:** 10.3390/ma16217034

**Published:** 2023-11-03

**Authors:** Haojie Luo, Yulei Du

**Affiliations:** School of Mechanical Engineering, Nanjing University of Science and Technology, Nanjing 210094, China; luohj_njust@njust.edu.cn

**Keywords:** additive manufacturing, laser powder bed fusion, bulk metallic glasses, mechanical properties, microstructure

## Abstract

Bulk metallic glasses (BMGs) display excellent strength, high hardness, exceptional wear resistance and corrosion resistance owing to its amorphous structure. However, the manufacturing of large-sized and complex shaped BMG parts faces significant difficulties, which seriously hinders their applications. Laser powder bed fusion (LPBF) is a typical additive manufacturing (AM) technique with a cooling rate of up to 10^8^ K/s, which not only allows for the formation of amorphous structures but also solves the forming problem of complex-shaped BMG parts. In recent years, a large amount of work has been carried out on the LPBF processing of BMGs. This review mainly summarizes the latest progress in the field of LPBF additively manufactured BMGs focusing on their mechanical properties. We first briefly review the BMG alloy systems that have been additively manufactured using LPBF, then the mechanical properties of LPBF-fabricated BMGs including the micro- and nano-hardness, micropillar compressive performance, and macro-compressive and tensile performance are clarified. Next, the relationship between the mechanical properties and microstructure of BMGs produced via LPBF are analyzed. Finally, the measures for improving the mechanical properties of LPBF-fabricated BMGs are discussed. This review can provide readers with an essential comprehension of the structural and mechanical properties of LPBF-manufactured BMGs.

## 1. Introduction

Bulk metallic glasses (BMGs) are without argument one of the most attractive metallic material systems, as they successfully combine the best qualities of metal and glass, including an outstanding hardness and strength [1,2,3], high elasticity limit [4], high abrasion and corrosion resistance [5,6], as well as wonderful magnetic [7,8] and catalytic properties [9,10]. BMG has been used in the production of many products, such as sports goods, watch parts, electromagnetic casings, optical parts, decorative parts, chokes, power inductors, magnetic field recognition systems, electromagnetic wave shields, and micro gear motors [11,12,13]. The excellent performance of BMG is related to its unique amorphous structure [14], which makes it free from defects like dislocations, grain boundaries, and chemical segregation of conventional crystalline alloys [15,16].

From a historical perspective, the evolution of metallic glass (MG) has always been strongly supported by new manufacturing technologies. In 1960, Duwez et al. [17] prepared Au_75_Si_25_ metallic glass for the first time by a rapid quenching technique, known as melt spinning, which is capable of cooling the molten alloy at a very high rate of 10^5^–10^6^ K/s. For a long time afterwards, however, MGs can only be prepared into very small-sized powders or stripes [18]. Multicomponent BMGs with large supercooled liquid zones and near-deep eutectics were designed [19], and several types of BMGs have been obtained, including Pd- [20], Pt- [21], Fe- [22], and Zr- [23], as well as Ti- [24], Ni- [25], Cu- [26], Mg- [27], and La-based alloys [28]. However, so far, most BMGs are still less than 30 mm, which is far from the actual size of the structural material. BMGs are usually processed directly through conventional copper mold casting and quenched quickly from the melt to achieve the required cooling rate to avoid crystallization. Although the copper mold casting method allows for simultaneous cooling and molding in a single step, it is ultimately difficult to achieve complex-shaped BMG parts due to limitations in mold size and cooling rate. In addition, owing to its tremendous strength and toughness, BMG exhibits poor processability at room temperature. Therefore, BMG is a typical hard-to-form material [29]. Thus, how to break through the limitations of BMGs in terms of the size and geometric shape remains the key to expanding their applications.

**Figure 1:**
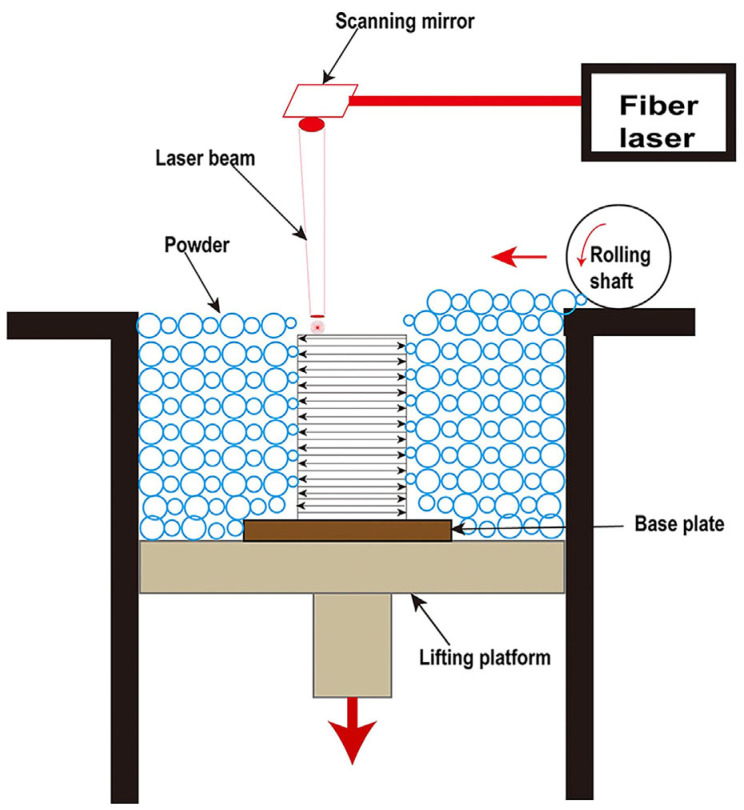
Schematic of the LPBF process [35].

For the past few years, additive manufacturing (AM) technology has been developing rapidly as a new technology, which involves the formation of highly complex components without the need of a mold [30]. Due to its characteristics of a high cooling rate and layer-by-layer processing, the AM technique allows the simultaneous manufacturing of large-sized and complex-shaped BMG parts [31,32]. To date, laser powder bed fusion (LPBF), direct energy deposition (DED), laser solid forming (LSF), and laser foil printing (LFP) have been employed for the additive manufacturing of BMG parts. Among them, LPBF is the most widely used AM technology because it has the advantages of a high cooling rate and high forming accuracy [33]. As shown in Figure 1, LPBF works by melting a bed of particles selectively, depending on a CAD model that was previously deposited on a removable substrate by means of a roller. The part is then reduced by one layer’s thickness (typically 10–100 μm) through the lifting platform and the process is repeated until the entire part is fully printed [34,35]. Due to the high scanning speed, the small laser beam diameter, the high laser power, and the short interaction time between the powder and the laser, a maximum localized cooling rate (R_c_) of 10^8^ K/s can be achieved [33]. Therefore, the LPBF process does have a higher cooling rate than the critical cooling rate required for the formation of most BMG glass systems [36]. As a result, the metallic glass can be easily achieved using LPBF [37]. The successful fabrication of Fe-based MG scaffolds with relatively complex geometries using LPBF was firstly reported in 2013 by Pauly et al. [38], which confirmed the feasibility of the method of preparing BMGs using LPBF technology. Since then, a significant amount of study has been undertaken on the LPBF production of BMGs.

It is well-known that BMG is known for its superior mechanical properties, which are the foundation of BMG as an advanced structural material. So far, various BMGs, including Zr-, Fe-, Cu-, etc. alloys, have been manufactured through the LPBF method. However, these studies indicate that the strength of the LPBF-printed BMGs is significantly weaker than that of their corresponding as-cast amorphous counterparts. The reason for this is still an unresolved issue. In view of the large number of related literature reports, it is necessary to review the research on the mechanical properties of LPBF-fabricated BMGs in order to enhance the understanding of the current state of the research and to facilitate the progress of mechanical property optimization. Therefore, this review aims to summarize the currently available mechanical data on LPBF additively manufactured BMGs to paint a state-of-the-art picture of the present literature. This review will also analyze the influence of defects on the mechanical characteristics and possible measures to improve the mechanical properties of LPBF-fabricated BMGs.

## 2. BMGs Fabricated using LPBF

BMGs are a family of multicomponent alloys, and a range of alloying systems like Zr-, Ti-, Fe-, Cu-, Mg-, and La-based alloys have been developed through conventional processing. However, only a few BMG alloys, mainly in Zr-, Fe-, Al-, Cu-, and Ti-based glass systems, have been prepared using LPBF due to the difficulty in obtaining the required BMG powders for printing. Table 1 summarizes the BMGs that have been fabricated using LPBF in recent years and their structural state.

### 2.1. Zr-Based BMG

**Figure 2:**
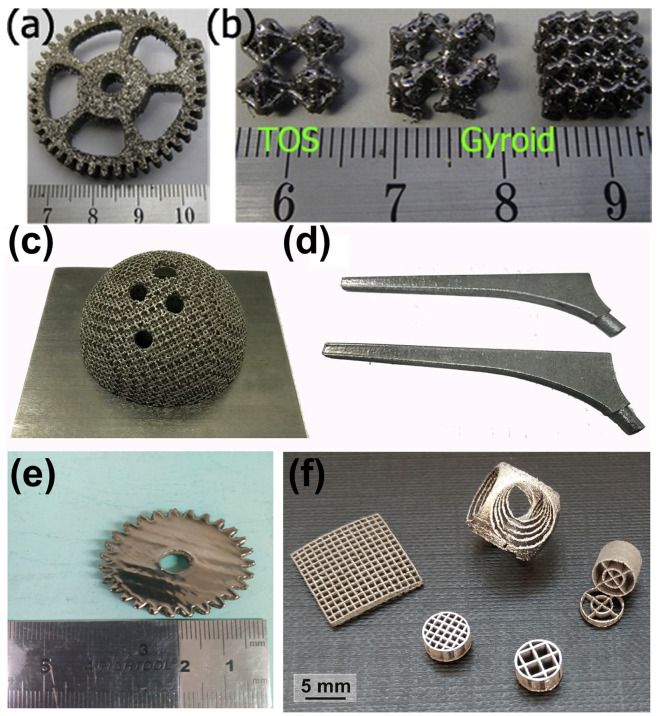
Amorphous Zr_52.5_Cu_17.9_Ni_14.6_Al_10_Ti_5_ BMG parts manufactured using LPBF: (**a**) gear sample; (**b**) TOS structure and two gyroid structures [39]. Amorphous Zr_60.14_Cu_22.31_Fe_4.85_Al_9.7_Ag_3_ BMG components produced using LPBF for biomaterial applications: (**c**) acetabular cup; (**d**) femoral prosthesis [46]. (**e**) Amorphous Zr_60_Fe_10_Cu_20_Al_10_ BMG gear sample (diameter 35 mm and height 5 mm) [48]. (**f**) Amorphous Zr_52.5_Ti_5_Al_10_Ni_14.6_Cu_17.9_ BMG scaffolds produced using LPBF [40].

Zr-based BMGs have an excellent glass forming ability (GFA), and using copper mold casting can easily obtain BMG rods with a diameter larger than 10 mm [65]. This characteristic also enables Zr-based alloys to exhibit excellent LPBF formability. In 2016, a Zr-based BMG with a composition of Zr_52.5_Ti_5_Cu_17.9_Ni_14.6_Al_10_ was successfully manufactured through LPBF. Since then, the LPBF of Zr-based BMGs has attracted a lot of attention [40,41,42,43,44,45,46,47,48,49]. The most common alloy among them was Zr_59.3_Cu_28.8_Al_10.4_Nb_1.5_, also known as AMZ4. However, since AMZ4 is a commercial alloy powder, it usually includes more impurities. Therefore, AMZ4 BMG requires more work to form an amorphous phase than other Zr-based amorphous alloys [44,45]. Figure 2 shows some Zr-based BMG components manufactured through LPBF. This demonstrates the ability of LPBF to manufacture complex-shaped Zr-based BMG components, which is challenging to do using conventional casting methods.

### 2.2. Fe-Based BMG

**Figure 3:**
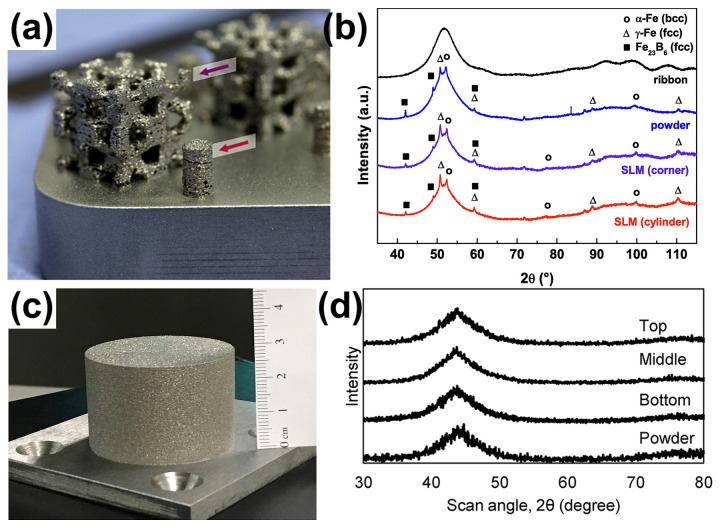
(**a**) The 3D scaffold structure of partially crystallized Fe_74_Mo_4_P_10_C_7.5_B_2.5_Si_2_, where the arrows indicate the position of the X-ray diffraction measures of the samples; (**b**) XRD patterns of a corner of the scaffold and the cylinder, showing obvious crystal phases [38]. (**c**) An FeCrMoCB BMG cylinder with a size of Ø 45 × 30 mm, (**d**) XRD of FeCrMoCB cylinders (bottom, middle, and top) [49].

The GFA of the first Fe-based MGs was relatively bad, and their critical sizes were too small, making them difficult to form and process. Previous studies have found that the addition of C and B elements to Fe-based alloys can enhance their GFA, and the large Fe-based BMG components have been successfully fabricated [66]. In addition to their unique mechanical properties, Fe-based BMGs have exceptional magnetic properties. These properties are attributed to the absence of crystal-related defects in BMGs. The Fe-based BMG’s soft magnetic characteristics are diminished by crystallization, therefore requires a high amorphous structural state [67]. It is obvious that the traditional copper mold casting technology is difficult to prepare large-sized Fe-based BMGs with fully amorphous structures, and LPBF additive manufacturing technology provides an opportunity to overcome this limitation. In 2013, an Fe-based BMG (Fe_74_Mo_4_P_10_C_7.5_B_2.5_Si_2_) was prepared using LPBF [38]. Although the parts obtained using LPBF were partially crystallized with visible defects such as porosity and interlayer cracks as shown in Figure 3a,b, this preliminarily confirms the feasibility of LPBF in manufacturing Fe-based BMGs. Afterwards, a number of Fe-based BMGs with various chemical compositions have been generated using the LPBF method, as listed in Table 1 [35,49,50,51,52,53,54,55]. Remarkably, Mahbooba et al. [49] prepared a fully amorphous Fe-based BMG with a composition of Fe_37.5_Cr_27.5_Mo_10_C_12_B_13_ using LPBF, with a diameter of 45 mm, which is twice as large as that of casting, as shown in Figure 3c,d.

### 2.3. Al-Based BMG

**Figure 4:**
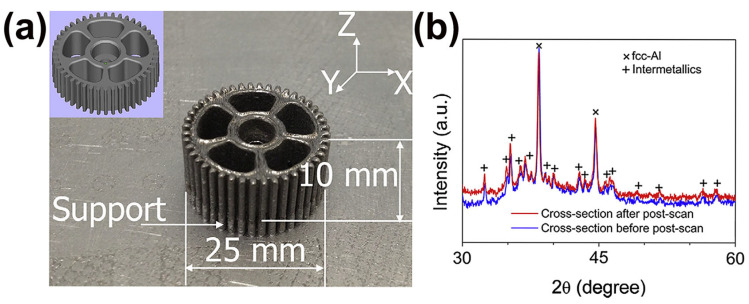
(**a**) Fully crystallized Al_85_Ni_5_Y_6_Co_2_Fe_2_ gear prepared using LPBF, with CAD model of the gear in the upper left corner; (**b**) corresponding XRD pattern showing obvious crystallization peaks [58].

Al-based BMGs have an excellent strength-to-weight ratio and corrosion resistance. However, the GFA of Al-based alloys is very bad, which makes it difficult to prepare Al-based MGs in a completely amorphous state and with dimensions larger than 1 mm by conventional methods over a long period of time [68,69]. In 2014, Li et al. [58] utilized LPBF to prepare an Al_85_Ni_5_Y_6_Co_2_Fe_2_ BMG, however, the as-built sample clearly crystallizes, forming an amorphous matrix composite material, as shown in Figure 4. Subsequently, they successfully prepared Al_86_Ni_6_Y_4.5_Co_2_La_1.5_ with an almost fully amorphous structure by carefully adjusting the LPBF processing parameters [57]. So far, three Al-based BMGs, namely, Al_86_Ni_6_Y_4.5_Co_2_La_1.5_ [57], Al_85_Nd_8_Ni_5_Co_2_ [59], and Al_85_Ni_5_Y_6_Co_2_Fe_2_ [58], have been prepared using the LPBF method.

### 2.4. Cu-Based BMG

**Figure 5:**
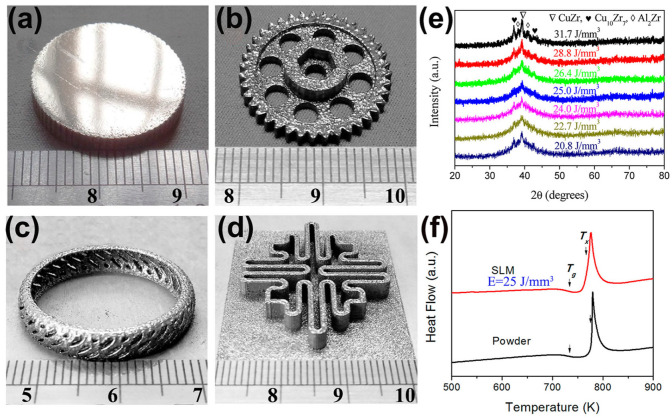
(**a**–**d**) Some amorphous Cu_50_Zr_43_Al_7_ parts with large sizes and complex shapes, which were produced with the optimal LPBF process parameters (E = 25 J/mm^3^); (**e**) XRD plots of different energy densities, where the samples with E = 25 J/mm^3^ are almost completely amorphous; (**f**) DSC plots of the optimized sample and the original powder, which shows the typical features of amorphous phase, where the arrows point to the glass transition temperature (T_g_) and the crystallization onset temperature (T_x_) [61].

Cu-based BMG is known for its low cost and high strength [70]. The Cu–Zr–Al system has the highest as-cast strength among the Cu-based BMGs, and its fracture strength is generally in the range of 1.6 GPa to 2.0 GPa. However, their GFA is low, with a critical casting diameter of only 3 mm. Their GFA can be further increased by adding more elemental components. In 2019, a ternary Cu-based BMG, Cu_50_Zr_43_Al_7_, was prepared for the first time using LPBF technology [61]. Some large and complex amorphous parts were obtained using the optimal process parameters of LPBF (E = 25 J/mm^3^), as shown in Figure 5.

### 2.5. Ti-Based BMG

**Figure 6:**
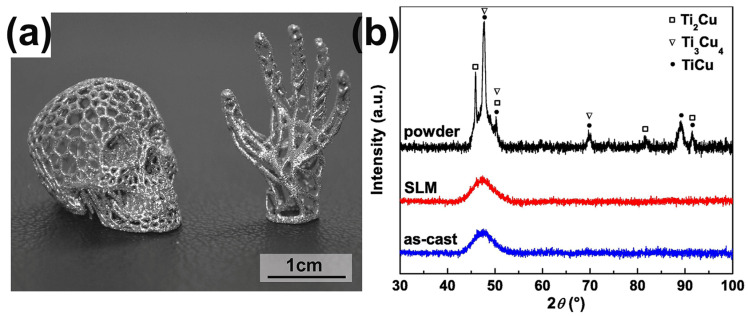
(**a**) Amorphous and complex structural Ti_47_Cu_38_Zr_7.5_Fe_2.5_Sn_2_Si_1_Ag_2_ components made using LPBF. (**b**) The corresponding XRD patterns of cylindrical LPBF-fabricated samples and cast rods. Crystal peaks only occur in gas-atomized powders [56].

Ti-based alloys are employed in a wide range of applications such as aerospace, orthopedics and dental implants, owing to their great biocompatibility and strength-to-weight ratio. However, the research on Ti-based BMGs has not been deep enough, and few Ti-based BMG systems have been successfully developed. Although the addition of the element beryllium can significantly increase the GFA of Ti-based alloys, leading to a critical casting diameter of more than 14 mm. Unfortunately, beryllium is a toxic element, so its application is limited to a certain extent. In 2015, a Ti-based BMG (Ti_47_Cu_38_Zr_7.5_Fe_2.5_Sn_2_Si_1_Ag_2_), free of beryllium and nickel elements, was successfully developed with a critical casting diameter of 7 mm [71]. In 2018, Deng et al. [56] produced a Ti-based BMG (Ti_47_Cu_38_Zr_7.5_Fe_2.5_Sn_2_Si_1_Ag_2_) through LPBF. The as-built sample not only achieved a relative density of 99.5% to 99.7% but also presented a completely amorphous structure, as shown in Figure 6. This study indicates that LPBF has important value in the manufacturing of biomedical Ti-based BMG components.

### 2.6. Precious BMG

**Figure 7:**
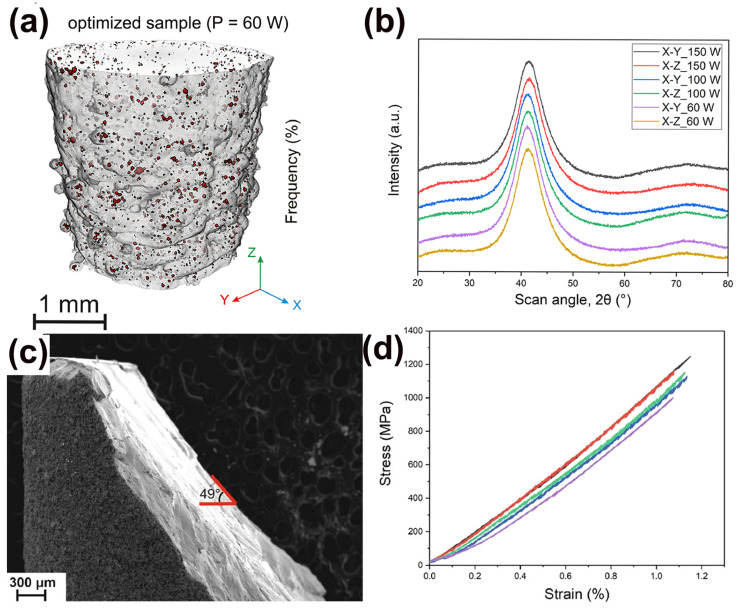
(**a**) The μ-CT image of Pd-based BMG prepared using optimized LPBF parameters (P = 60 W), exhibiting no cracks and high densities, with a porosity of only 0.4%; (**b**) XRD patterns from two cross-sections of printed samples, X-Y and X-Z; (**c**) Fracture morphology of the optimized cylinder; (**d**) compression stress–strain curve, showing excellent compressive strength [64].

Precious metals have numerous applications due to their metallic luster and superior mechanical characteristics like hardness and wear resistance, especially in commercial areas such as the watch and jewelry industry [72,73]. Usually, the mechanical performances of precious BMGs are higher than the performances of their crystalline alloy counterparts. Therefore, it makes sense to design and develop precious BMGs to meet the needs of the watch and jewelry industry [74,75]. So far, BMGs based on various precious metals have been developed, like Pd-based [76], Pt-based [77], and Ag-based BMGs [78]. Recently, a fully amorphous Pd-based BMG (Pd_43_Cu_27_Ni_10_P_20_) was successfully manufactured using the LPBF method [64]. The as-printed Pd-based BMG cylindrical samples were dense and crack-free, and also showed exceptional mechanical performance, like high hardness and compressive strength, as shown in Figure 7.

## 3. Mechanical Properties of LPBF-Fabricated BMGs

### 3.1. Microhardness

It is well-known that hardness is an important mechanical property of a material, and therefore the hardness of a material is required to be tested [79]. Hardness testing is most commonly used for BMGs manufactured by the LPBF process. Table 2 summarizes the microhardness results of BMGs currently produced using LPBF. For example, Shi et al. [80] tested the hardness of AMZ4 and found the BMG made using LPBF with porosity as low as 0.45% had a macroscopic hardness of 484 HV5, which reaches the hardness of the corresponding cast BMG. Sohrabi et al. [36] conducted microhardness tests on AMZ4 BMG made using LPBF, and even though the BMG contained nanocrystals, a high hardness of 446 HV5 was achieved. Currently, the difference in the microhardness between BMG made using LPBF and traditional cast BMG can be almost ignored [46,81,82]. Zhang et al. [63] found that the microhardnesses of the solidified molten pool and HAZ were 593.4 ± 23.8 and 462.2 ± 18.6 HV0.05, respectively, which indicated that the solidified molten pool was harder than the HAZ.

**Figure 8:**
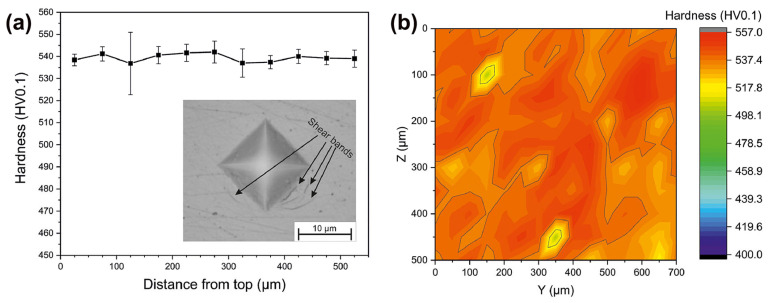
(**a**) Microhardness results (HV0.1). (**b**) Hardness map obtained from 165 indentations [64].

In addition, Sohrabi et al. [64] employed a smaller force to study the variation of hardness with the depth of LPBF-fabricated BMG samples and found that the hardness of LPBF-fabricated Pd-based BMG samples was almost uniform throughout the thickness up to 539 HV0.1, indicating a homogeneous and high densification of the structure, as shown in Figure 8.

### 3.2. Nanohardness

**Figure 9:**
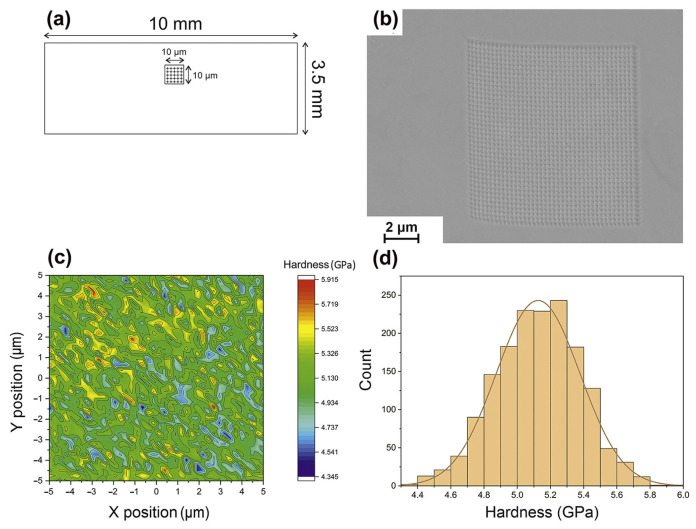
(**a**) Schematic of the nanoindentation test locations of LPBF-fabricated AMZ4 BMG samples. (**b**) SEM of 1600 indentations performed. (**c**) The 10 × 10 μm^2^ nanohardness testing area. (**d**) A histogram displaying the Gaussian distribution of hardness [36].

The detection of heterogeneity in the structure of BMGs fabricated via LPBF is usually revealed through the use of nanohardness maps [33,36,39,84,85]. For example, Li et al. [39] revealed the heterogeneity of LPBF-fabricated Zr_52.5_Ti_5_Cu_17.9_Ni_14.6_Al_10_ BMG samples using nanohardness mapping with a maximum average hardness of about 6.5 GPa. Sohrabi et al. [36] conducted the nanoindentation testing of AMZ4 manufactured through LPBF, and the average value was 5.13 ± 0.25 GPa, as shown in Figure 9. The hardness is relatively uniform overall, and the red spots are due to nanocrystal precipitation.

Compared with BMG manufactured using traditional technology, the amorphous phase hardness of BMG manufactured with LPBF is relatively higher [86]. Fe_55_Cr_25_Mo_16_B_2_C_2_ BMG prepared using LPBF, has a nanohardness of 14 GPa, equivalent to 1260 HV [52]. During the nanoindentation test of LPBF-fabricated {(Fe_0.6_Co_0.4_)_0.75_B_0. 2_Si_0.05_}_96_Nb_4_ BMG, Luo et al. [84] found that the amorphous content decreased with the increase of the LPBF laser energy input, and the hardness increased, with a maximum nanohardness of 16.9 ± 1.1 GPa [84].

Li et al. [58] investigated the effect of the scanning strategy on the hardness of Al_85_Ni_5_Y_6_Co_2_Fe_2_ BMGC. The 1.41 GPa hardness after rescanning was higher compared to the 1.07 GPa hardness after a single scan. This is explained by the reality that rescanning may decrease the free volume of the material, thus increasing the hardness of the BMGC [87]. In addition, the nanoindentation technique can also measure the hardness of the solidified molten pool and heat-affected zone [35,45,88,89,90].

### 3.3. Micropillar Compression Behavior

**Figure 10:**
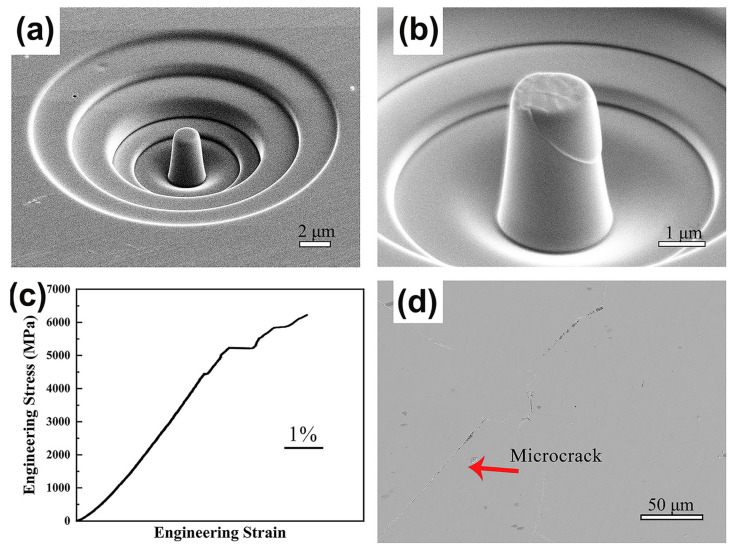
(**a**,**b**) SEM images of LPBF-fabricated Fe_55_Cr_25_Mo_16_B_2_C_2_ BMG micropillars in compression tests; (**c**) stress–strain curves; (**d**) cross-sectional microscope images of BMGs [52].

Size effects have been observed in BMGs manufactured using LPBF technology [91]. The properties of the amorphous structure of the BMG itself can be tested during compression tests on BMG microcolumns prepared using LPBF by ignoring the defects introduced and crystallization initiated during the layer-by-layer additive manufacturing process [52,82,92]. Therefore, the compressive strength of a micropillar specimen of Fe_55_Cr_25_Mo_16_B_2_C_2_ prepared using LPBF can reach 6000 MPa, which is more consistent with the BMG material’s theoretical strength [52], as shown in Figure 10.

### 3.4. Compressive Behavior

The most studied mechanical property in BMG manufactured through LPBF is compressive strength. Table 3 summarizes the composition, strength, plasticity, and sample size of different systems of BMGs for LPBF printing reported in recent years. It can be seen that the fracture strength of various 3D-printed BMGs ranges from 100–1800 MPa, while the compressive plastic strain ranges from only 0–3.17%. The strength of Zr- and Ti-based BMGs formed by 3D printing is above 1500 MPa, but their maximum compressive plasticity is only 2.15% [43]. Ouyang et al. [41] compared the compressive fracture strength of 3D-printed Zr_55_Cu_30_Al_10_Ni_5_ BMG rods with cast rods. The strength and Young’s modulus of LPBF-fabricated Zr_55_Cu_30_Al_10_Ni_5_ BMG were relatively weaker owing to the presence of pores, but still maintained a sufficiently high yield strength of 1504 MPa. For LPBF-fabricated Fe- and Al-based BMGs, the strengths were below 200 MPa, caused by significant brittleness and the existence of microcracks [93]. During the LPBF process, the Al-based BMG printed sample cracked severely, making it impossible to conduct mechanical property tests [57]. From Table 3, it can be seen that so far, the majority of studies have concentrated on Zr- and Fe-based BMG. The research on laser powder bed fusion forming of other BMGs, like Ti- or Cu-based systems, has been limited due to their low GFA or poor machinability.

In terms of plasticity, the majority of LPBF-fabricated BMGs that have been documented thus far exhibit negligible or no compression plasticity at ambient temperature, as is the case with other methods of producing BMGs. The 3D-printed Zr_60.14_Cu_22.31_Fe_4.85_Al_9.7_Ag_3_ BMG also exhibits a strong dimensional impact, similarly to the casting condition [98]. When the LPBF-fabricated sample’s size was shrunk from 3 mm to 1 mm, the plastic strain increased by about three times [46]. This may be due to the fact that the smaller the size of the sample, the fewer defects are formed by the LPBF.

Deng et al. [56] studied the mechanical performances of an LPBF-fabricated Ti-based BMG (Ti_47_Cu_38_Zr_7.5_Fe_2.5_Sn_2_Si_1_Ag_2_) and found that its fracture strength could reach up to 1690 MPa, but the plasticity remained zero. It displayed close to a 90° fracture angle, indicating a brittle fracture. Since the LPBF-fabricated Ti_47_Cu_38_Zr_7.5_Fe_2.5_Sn_2_Si_1_Ag_2_ BMG samples have a completely amorphous structure in the X-ray diffraction range, a porosity of about 1.5% may be the reason for the premature part failure. Similarly, in the case of Cu-based BMGs, there is a significant brittleness like most other BMG systems. As a result, LPBF-fabricated Cu_50_Zr_43_Al_7_ BMG also exhibits zero plasticity under compressive loading and a low strength of 1044 MPa, which may also be attributed to the presence of porosity [61].

Paul et al. [40] performed a room temperature compression test of LPBF-fabricated and cast Zr_52.5_Cu_17.9_Ni_14.6_Al_10_Ti_5_ BMG samples. The results show that the strength of LPBF-fabricated BMG samples is 1670 MPa, which is lower than the casting strength of 1780 MPa, which may be caused by defects such as pores and unmelted powder formed during the LPBF process. Two intersecting inclined fracture surfaces can be observed in the LPBF-fabricated BMG compression specimens, which suggests that the fracture process is different to that of pure shear fractures. Deng et al. [94] found that the fracture strength of the LPBF-fabricated cylindrical specimens was about 1710 ± 40 MPa, while the fracture strength of the LPBF-fabricated rectangular specimens was about only 1420 ± 20 MPa. Both values were significantly lower than the strength of the corresponding castings, and the pores serve as stress concentrators in the material during mechanical loading.

**Figure 11:**
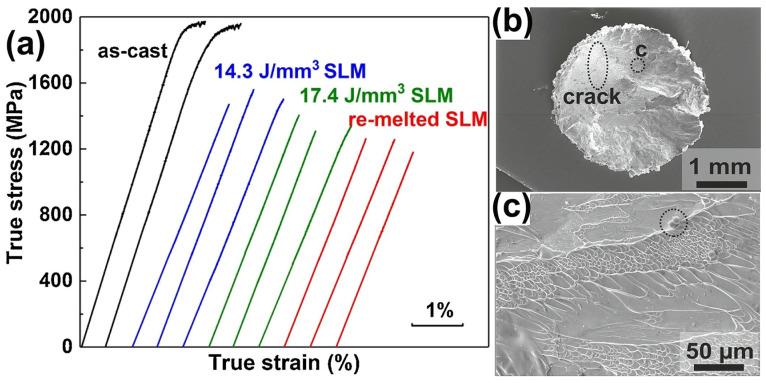
(**a**) Compressive stress–strain curves of as-cast and LPBF-fabricated samples, where the E = 14.3 J/mm^3^ sample has the highest fracture strength of 1560 MPa, but is still lower than the corresponding cast sample; (**b**) fracture surface of LPBF-fabricated samples with E = 14.3 J/mm^3^, where a clear crack is observed; (**c**) higher magnification image of region in (**b**), which shows the presence of unmelted powder [62].

Prashanth et al. [59] prepared a high-strength Al_85_Nd_8_Ni_5_Co_2_ BMGC sample using LPBF. Under a room temperature compression test, Al_85_Nd_8_Ni_5_Co_2_ BMGC showed an ultimate compressive strength of 1.08 GPa, and a plastic strain of 2.45%. However, there are no other reports on the performance tests of Al-based BMGs successfully prepared using LPBF. Deng et al. [62] employed LPBF to fabricate a Cu_46_Zr_46_Al_8_ BMG and no plastic strain was observed, irrespective of varying the process parameter conditions, and the fracture strength was 1560 MPa, but still lower than that of the corresponding cast sample, as shown in Figure 11.

It was also found that the loading direction has a noteworthy influence on the fracture strength of LPBF-fabricated BMG specimens, which may be related to the layered structure obtained with 3D printing. Gao et al. [60] tested the compressive properties of an LPBF-fabricated Cu_46_Zr_47_Al_6_Co_1_ BMG and found that the specimens loaded perpendicular to the layer had a fracture strength of 0.93 GPa, more than those loaded parallel to the layer, which had a fracture strength of 0.67 GPa.

### 3.5. Tensile Behavior

**Figure 12:**
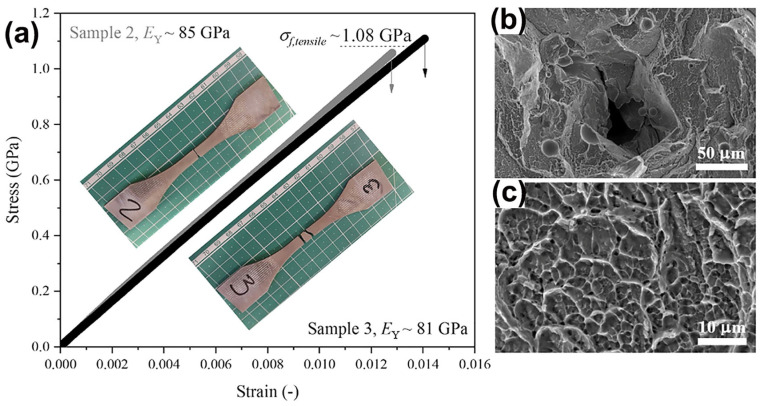
(**a**) Tensile test results of AMZ4 BMG fabricated using LPBF with a maximum strength of 1080 MPa; (**b**,**c**) SEM images of the fracture surface, where (**b**) shows lack of fusion defects and unmelted powder particles, and (**c**) shows a dimple pattern [101].

There has been very little research into the tensile properties of LPBF-prepared BMGs, with only three studies reported so far, and all of them were carried out on the industrial-grade alloy Zr_59.3_Cu_28.8_Al_10.4_Nb_1.5_ (AMZ4) BMG [99,100,101]. Best et al. [101] conducted tensile tests on LPBF-fabricated AMZ4 BMG, and the samples underwent a typical brittle fracture with a fracture strength of only 1.08 GPa, which is considerably lower than the compressive strength of the material of 1.8 GPa [82], as shown in Figure 12. The loss in the tensile strength may be attributed to the presence of defects in the samples. Shi et al. [99] used LPBF to create three different sets of uniaxial tensile samples with varying porosity levels, all of which fractured catastrophically under tension, with negligible macroscopic plasticity observed. The highest fracture strength measured was 1326 MPa. Sohrabi et al. [100] investigated the tensile performance of LPBF-fabricated AMZ4 BMG samples, and a high fracture strength of 1180 MPa was obtained, although no macro plastic deformation was detected in the tensile tests.

## 4. Effect of Defects on Mechanical Properties of LPBF-Fabricated BMGs

Unlike conventional copper mold casting, the LPBF process is based on layer-by-layer fabrication, which usually produces more defects during the manufacturing process, thus seriously affecting the mechanical performances of structural components [102]. Therefore, it is crucial to deeply understand the formation mechanism of defects in amorphous alloys during laser 3D printing, which is a prerequisite for developing printing strategies to reduce or eliminate 3D printing defects. These defects are divided into the following categories: porosity, LoF, microcracks, crystallization, and HAZ, which are described in more detail below.

### 4.1. Porosity

**Figure 13:**
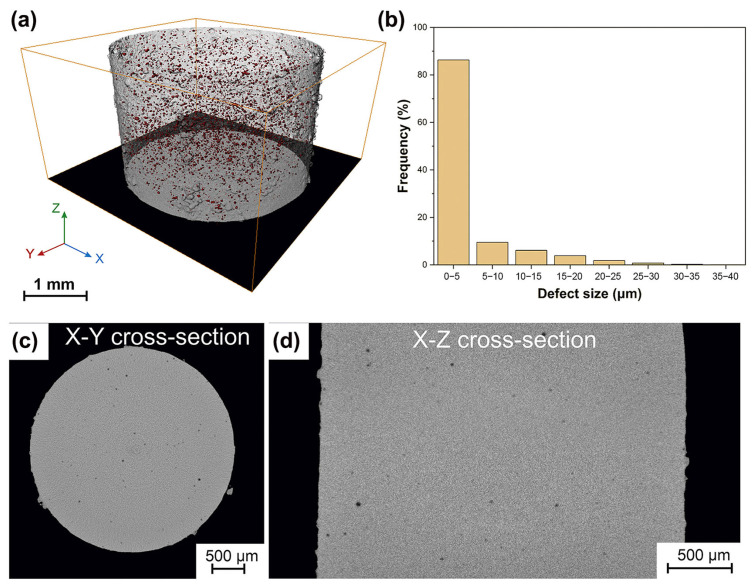
(**a**) 3D spatial distribution and morphology of porosity defects inside the cylinder revealed by μ-CT images; (**b**) the size distribution of the porosity defects; snapshots of the X-Y (**c**) and X-Z (**d**) cross-sections, with the porosity flaws displayed in black [36].

The most common issues are porosity defects, which significantly affect the mechanical performance of BMG parts produced with LPBF technology. Porosity resulting from the evaporation of volatile or low melting point materials in the molten pool, and porosity created by unstable molten pool collapse in the keyhole molten pool mode are the two primary causes of porosity during the LPBF production of BMG [103,104]. For example, Qiu et al. [102] found that the appropriate scanning speed and laser power are favorable to reduce the porosity; an increase in the scanning speed leads to an increase in porosity, which is due to the instability of the flow of the molten pool and the splashing of the molten material at a high scanning speed. Sohrabi et al. [36] fabricated an AMZ4 BMG cylinder with a diameter and height of 3 mm using LPBF to employ for μ-CT analysis. The three-dimensional distribution of the porosity defects inside the sample is shown in Figure 13, with most of the porosity defects (86%) ranging from 0 to 5 μm in size.

Due to the limited GFA of the BMG, the temperature cooling rate is more demanding, requiring faster scanning speeds as well as smaller energy inputs. As a result, the lack of energy input can be prone to a porous BMG and the incomplete melting of the powder. Porosity has been found in many LPBF studies on the preparation of metallic glasses [35,38,51,55,64,84,99]. Nong et al. [35] and Jung et al. [51] found that LPBF-fabricated Fe-based BMG samples prepared with a low energy density had a highly porous microstructure. Therefore, for the preparation of BMG through LPBF, the presence of pores is one of the main formation problems faced when preparing large-sized samples. Porosity is a factor that can lead to the premature failure of BMG parts manufactured using LPBF. Shi et al. [99] concluded that the porosity inherent in the LPBF process is considered to be a key factor affecting the mechanical integrity of AMZ4 BMG. They found that the ultimate tensile strength showed a rapid linear increase as the porosity decreased from 5.24% to 0.26%, and the tensile strength increased by 26% to 1326 MPa. Sohrabi et al. [64] found that 0.4% porosity was still present in the Pd-based BMGs generated using the optimized LPBF parameters through μ-CT experiments, which could be responsible for the 14% lower compressive strength compared to the cast samples.

### 4.2. LoF

Lack of fusion (LoF) defects are caused by insufficient molten metal to completely fill gaps during the LPBF process. Generally, it is believed that if the laser energy input is too low, it will result in a reduction of the molten pool width, allowing insufficient overlap between single-track melting pools, leaving unmelted powder. During the melting of the next powder layer, the low laser energy input fails to penetrate the powder layer, making it difficult to remelt the remaining unmelted powder in the upper layer, resulting in the formation of irregular keyholes, i.e., LoF defects [105].

**Figure 14:**
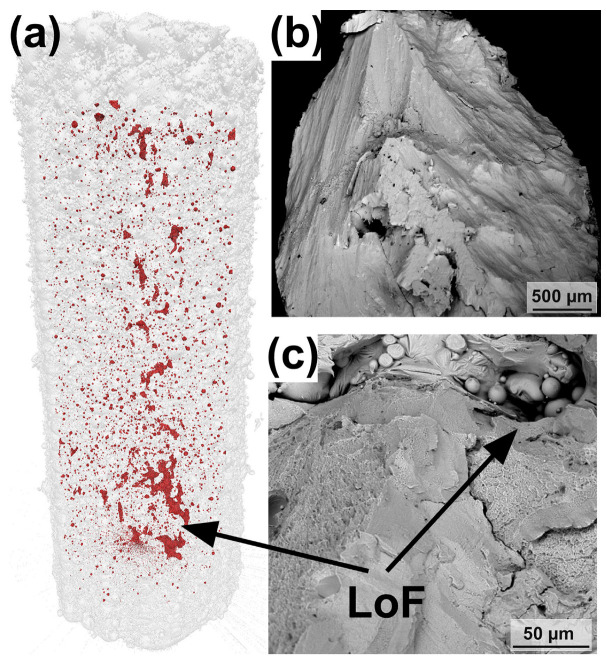
(**a**) The μ-CT of the LPBF-fabricated Zr-based BMG compression sample. (**b**,**c**) Fracture surfaces of the LPBF-fabricated BMG sample. Two intersecting fracture surfaces can be seen in (**b**), and unmelted powder particles were found in some larger LoFs in (**c**) [40].

Due to the fact that the shape of LoF is mostly irregular and its size can reach hundreds of micrometers, it may lead to stress concentration and damage mechanical properties [64]. Currently, several studies on the LPBF of BMG attribute LoFs to premature failure in tensile [100,101] and compressive [40] tests. Pauly et al. [40] reconstructed LPBF-prepared compression test specimens by μ-CT and found that the presence of large holes in the center of the specimens, i.e., LoF defects, shown in Figure 14a, which pass through several layers’ thicknesses and usually contain unmelted powder particles inside, may have contributed to the premature failure of LPBF-fabricated BMGs in compression tests compared to as-cast specimens. This is confirmed by the compression fracture morphology analysis shown in Figure 14b,c. Sohrabi et al. [100] reduced the LoF defects in the near-surface region by optimizing boundary processing parameters, such as laser power, resulting in a 28% and 27% increase in the impact toughness and tensile strength of LPBF-fabricated AMZ4 BMG, respectively. Although the increase of the laser power at the boundary and the core led to the precipitation of nanocrystals, the results show that the elimination of defects such as LoF is beneficial for improving the ultimate tensile strength of BMG. Best et al. [101] investigated the fracture surface of LPBF-fabricated AMZ4 BMG tensile specimens and found significant LoF defects. They associated the catastrophic failure of the specimens during tensile loading with LoF defects.

### 4.3. Microcracks

Microcracks are another common defect in 3D-printed BMG. During the LPBF process, only a small portion of the material undergoes rapid heating/cooling cycles, which may lead to sharp temperature gradients, significant thermal fluctuations, severe shrinkage, and high thermal stress, making BMGs very prone to cracking [55]. The presence of cracks considerably deteriorates the mechanical behavior of LPBF-fabricated BMGs. The BMGs formed using LPBF reported between 2013 and 2023 exhibit varying degrees of cracking, with the brittle Fe- and Al-based BMG systems being more prone to the occurrence of cracks [35,38,41,49,50,51,53,55,57,93,106,107].

Paul et al. [38] found the occurrence of cracks in Fe-based BMG samples fabricated using LPBF due to the high cooling rate of LPBF and the limited ductility of Fe-based BMG. Subsequently, Jung et al. [51] investigated the effect of LPBF process parameters on the microstructure of an Fe_68.3_C_6.9_Si_2.5_B_6.7_P_8.7_Cr_2.3_Mo_2.5_Al_2.1_ BMG. They concluded that the microcracks in the samples were due to the high cooling rate inherent in LPBF leading to a large temperature gradient between the laser-processed layer and the melt-solidified layer, which generated excessive thermal stresses. Moreover, the limited ductility and fracture toughness of the Fe-based BMG eventually led to cracks. Hofmann et al. [50] explored the 3D printing formability of FeCrMoBC BMG by conducting approximately 400 separate construction experiments using different LPBF process parameters. Most LPBF-fabricated BMGs were too brittle and cracked to undergo meaningful mechanical testing, and only in very narrow processing windows can crack-free components be obtained.

Li et al. [93] analyzed the thermal stress distribution surrounding the MP of Fe-based BMG during the LPBF process by finite element modeling (FEM). The findings demonstrated that the front portion of the molten pool created a maximum compressive stress of around 1.5 GPa, whereas a spot in the back of the molten pool showed a maximum tensile stress of 0.5 GPa. In reality, these thermal stresses were lower than the investigated Fe-based BMG’s 3.5 GPa fracture strength. However, during the LPBF process, the unavoidable formation of micropores leads to a concentration of stresses with a maximum of 4.1 GPa, which is much higher than the fracture strength of the Fe-based BMG, making microcracking unavoidable. On the contrary, FEM was conducted by Ouyang et al. [41] on Zr_55_Cu_30_Ni_5_Al_10_ BMG with higher toughness. The maximum stress concentration surrounding a microporous hole was approximately 1.4 GPa, which is less than the 1.5 GPa fracture strength for Zr-based BMG. Based on this, they prepared low thermal stress crack-free Zr-based BMG samples using the LPBF technique, thus maintaining a sufficiently high yield strength of 1504 MPa.

**Figure 15:**
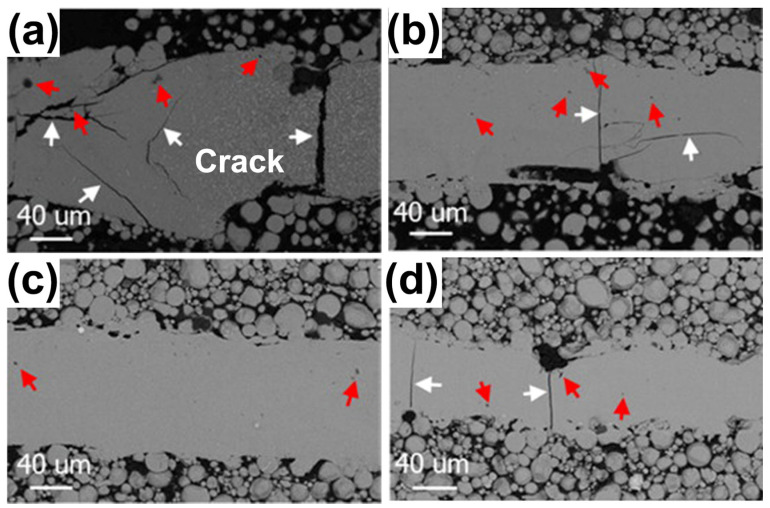
SEM-BSE images of X-Y sections of single-track molten pools fabricated using LPBF: (**a**) 200 W; (**b**) 160 W; (**c**) 120 W; and (**d**) 80 W. White arrows point to cracks and red arrows point to pores [57].

For the Al-based BMGs, Li et al. [57] found that it was hard to prepare crack-free Al_86_Ni_6_Y_4.5_Co_2_La_1.5_ BMGs using LPBF. Excessive laser power can cause excessive stress and lead to cracking, while a lower laser power would lead to an insufficient energy input and the powder particles could be incompletely melted, which would result in cracks and porosity, as shown in Figure 15. Therefore, Li et al. [58] used the remelting strategy as a stress-relief treatment method to prevent crack extension and successfully prepared Al-based BMG parts without visible cracks. This work demonstrated that the low-power rescanning strategy is beneficial for stress reduction and can prevent crack extension. However, for BMG systems with a lower GFA and toughness, more work is needed to reduce thermal stress.

From the above analysis, due to the inherently high cooling rate of LPBF, a larger temperature gradient is generated near the molten pool and at the interlayer bonding, which induces thermal stresses that may lead to microcrack formation. Therefore, Zr-based BMG systems with high fracture toughness are more likely to produce crack-free high-performance parts using LPBF.

### 4.4. Crystallization

Another challenge in the preparation of BMG using LPBF is the generation of crystallization defects due to the variation of thermal history during the printing process. As can be seen in Table 1, the presence of crystalline phases can be directly detected by XRD when the degree of crystallinity exceeds 10%. As mentioned earlier, in most cases, the crystallization of BMG is harmful and undesirable [38,61,62]. The mechanical properties of bulk metallic glass, especially the fracture stress, are very sensitive to its structural state. For example, Ouyang et al. [41] conducted compression tests on LPBF-fabricated Zr_55_Cu_30_Ni_5_Al_10_ BMGs and found that the fracture strength of 3D-printed Zr-based BMG was only 1504 MPa, which is significantly lower than its cast counterparts. They attributed this to the presence of brittle crystallization phases, which triggered a destructive brittle fracture in LPBF-fabricated BMGs.

### 4.5. HAZ

Owing to the uncontrolled crystallization that occurs in the heat-affected zone (HAZ), obtaining a monolithic glass remains a challenge for most BMG systems. In most cases, crystallization leads to an increased risk of cracking in LPBF-fabricated BMGs and adversely affects the densification and performance of the fabricated parts. Therefore, understanding the microstructure and formation mechanism of the HAZ is essential to enhance the mechanical properties of LPBF-printed BMGs. Current studies generally agree that the microstructure of LPBF-fabricated BMG samples consists of two main regions, the solidified molten pool and the HAZ [41,42,55,88]. Typically, the SMP consists of an amorphous phase, while the HAZ is partially crystalline (i.e., amorphous and crystalline phases coexist). In this case, the boundary between the fully amorphous and partially crystallized regions can be regarded as the edge of the HAZ.

**Figure 16:**
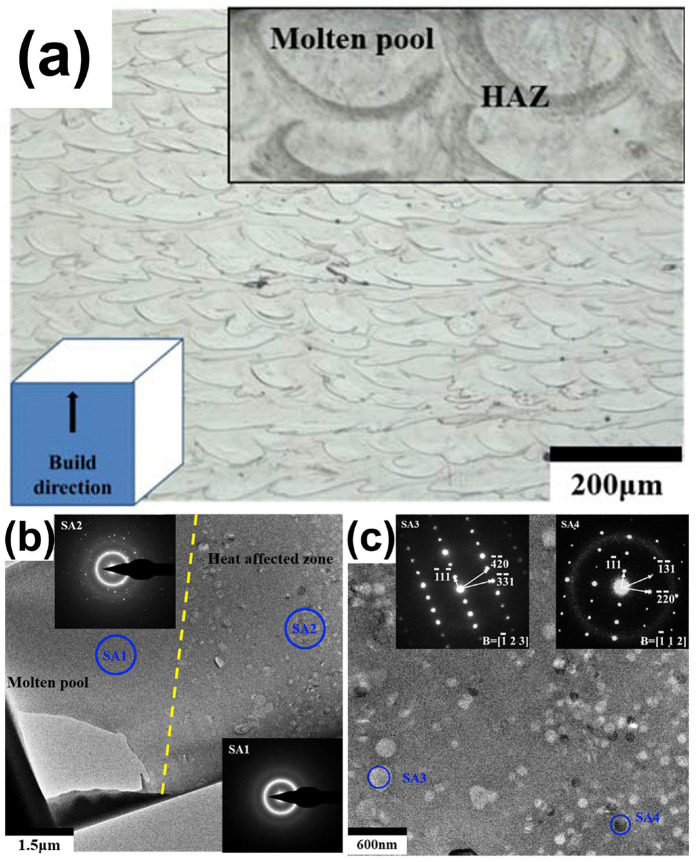
(**a**) Side view of 3D-printed Zr-based BMG revealing the distribution of the MP and HAZ; (**b**) TEM bright-field picture displaying the MP and HAZ borders; (**c**) diffraction pattern of selected locations in the HAZ [41].

Ouyang et al. [41] demonstrated the typical microstructure of LPBF-printed Zr-based BMG (Zr_55_Cu_30_Ni_5_Al_10_), as shown in Figure 16a, where the distributions of the solidified molten pool and the heat-affected zone can be clearly seen. They revealed that the region in the solidified molten pool (labeled with SA1) is completely amorphous, while the region in the heat affected zone (labeled with SA2) contains a mixed structure of amorphous phases and some nanocrystals with sizes of 100–200 nm, as shown in Figure 16b,c. Subsequently, FEM was introduced to understand the effect of the molten pool temperature distribution on crystallization, and it was found that the cooling rate in the MP was sufficiently high (8.2 × 10^4^ K/s) to be a monolithic glass; whereas the HAZ, due to reheating caused by the laser beam irradiation on the adjacent regions of the powder bed, had a temperature higher than T_x_, and thus was partially crystallized. Based on this, Ouyang et al. [55] continued LPBF forming of an Fe_43.7_Co_7.3_Cr_14.7_Mo_12.6_C_15.5_B_4.3_Y_1.9_ BMG and found that the solidified molten pool was always completely amorphous at various laser energy densities, and that excessive laser energy densities triggered extensive crystallization in the HAZ. Following an FEM analysis of the HAZ’s temperature field, it was discovered that while the cooling rate was high enough (4.37 × 10^4^ K/s) to inhibit crystallization, partial crystallization still happened there. This was actually due to laser flashes on the previously formed amorphous phase, which had a peak temperature higher than the crystallization temperature.

Li et al. [57] performed a single line scan of an Al_86_Ni_6_Y_4.5_Co_2_La_1.5_ BMG. It was found that the further away from the center of the solidified molten pool, the more severe the crystallization was. The width of the HAZ increased with increasing laser power and the crystallization became more severe. Although the cooling rate at a high laser power is fast, the crystallization of BMG increases. This indicates that the formation of the amorphous phase is not only affected by the cooling rate, but is also more easily affected by the HAZ in the process of forming the Al-based BMG using LPBF. Therefore, it is more important to consider the HAZ factor for the optimization of the process parameters during the LPBF of BMGs.

## 5. Measures for Improving Mechanical Properties of LPBF-Fabricated BMGs

So far, producing large-sized BMG samples without any cracks or pores through LPBF remains a challenge. In the past few years, many scholars around the world have overcome various defects in the preparation of BMG mainly through the optimization of LPBF process parameters, and the adjustment of scanning strategies [39,40,53,58,108,109], thereby improving its mechanical properties.

### 5.1. Optimization of LPBF Process Parameters

By optimizing LPBF process parameters, the formation of defects in the as-built BMG can be effectively suppressed. Therefore, the optimization of LPBF process parameters is an important factor in preparing BMG parts with excellent mechanical properties [38,51]. Table 4 summarizes the optimal process parameters reported in the literature that can achieve an amorphous structure in the as-built BMG parts.

It is known that the LPBF process parameters can have an overall effect on the cooling rate, thus resulting in different microstructural/mechanical properties. During the LPBF process, the energy density (E) is typically employed to define the laser energy input into the powder bed [110]:

E = P/vht(1)

where E is the energy density (J/mm^3^), P is the laser power (W), v is the scan speed (mm/s), h is the hatch spacing (mm), and t is the thickness (mm). The adjustment of the above processing parameters is usually performed during LPBF printing to customize BMG parts with suitable microstructural and mechanical properties.

The level of laser power used for LPBF is positively related to the energy input to the powder bed. Pauly et al. [40] reported that the porosity of LPBF-fabricated Zr-based BMG could be decreased through increasing the laser power. However, if the laser power is too high, the thermal oscillations in the molten pool are more violent, thus leading to a decrease in the GFA of the BMG and crystallization [51,57]. In addition, the molten pool can become both narrow and deep. Therefore, a reasonable heat input can widen the molten pool width and distribute the heat flow uniformly. For example, at a very high laser power (P ≈ 200 W), it is almost impossible to bypass the crystallization of Al_86_Ni_6_Y_4.5_Co_2_La_1.5_ BMG [57]. At a high laser power, severe crystallization occurs despite the high cooling rate; at a low laser power, the amorphous nature is preserved. This depends on the homogeneous or heterogeneous chemical distribution resulting from thermal fluctuations within the molten pool. kept the same Therefore, a reasonable laser power is needed to ensure the porosity and vitrification of the printed BMG parts during the preparation of BMG using LPBF.

**Figure 17:**
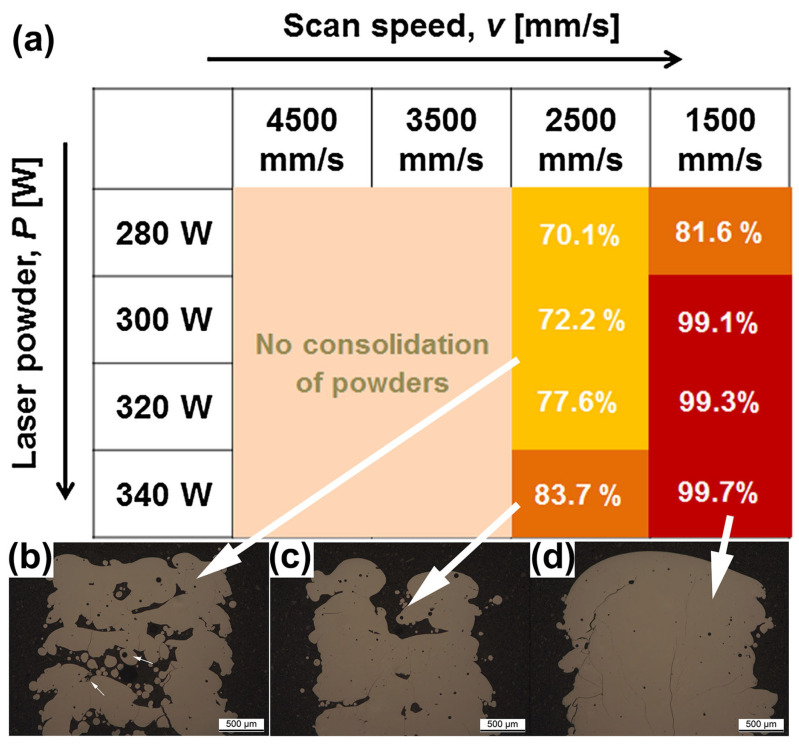
(**a**) Relative density plots of LPBF-fabricated Fe_68.3_C_6.9_Si_2.5_B_6.7_P_8.7_Cr_2.3_Mo_2.5_Al_2.1_ BMG samples versus process parameters, where the darker the color, the higher the densities. (**b**–**d**) OM images of LPBF-fabricated samples, where white arrows correspond to different densities [51].

The scanning speed during LPBF printing is one of the factors that affects the cooling rate. In order to prepare a high percentage of amorphous phase BMG parts, it is usually necessary to set a high scanning speed. For example, when the laser power is constant at 250 W and the scanning speed is 1000 mm/s, the glass volume fraction of Fe-based BMGs printed using LPBF can reach 98%, but when the scanning speed is reduced to 200 mm/s, the glass volume fraction is only 50% [55]. However, incorrect scanning parameters can lead to incomplete powder melting or even failure in MG manufacturing. Jung et al. [51] observed that when the laser scanning speed was too high (over 2500 mm/s), the input energy to the powder bed was insufficient and incompletely melted powder particles could be observed in the OM, as shown in Figure 17. At a scanning speed of 1500 mm/s, structures with densities greater than 99% were obtained.

The hatch spacing can affect the bonding between adjacent single-track molten pools. According to Pauly et al. [40], at the same laser intensity, a reduction in the hatch spacing increases the relative density of LPBF-fabricated Zr-based BMGs. However, decreasing the hatching spacing results in excessive local energy input, leading to massive crystallization [39]. Also, the layer thickness affects the amorphous content in LPBF-printed BMGs. Usually, the thicker the layer, the higher the amorphous content, but this also leads to more defects. Consequently, when producing BMGs using LPBF, an appropriate hatch spacing and layer thickness are needed.

**Figure 18:**
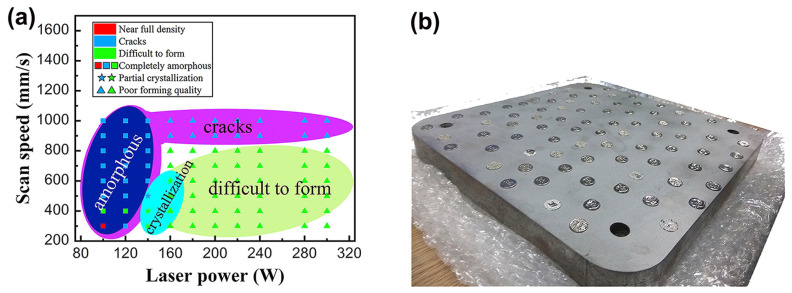
(**a**) LPBF processing maps displaying different feature regions. Different colors and markings are used to highlight areas processed by different laser powers and scanning speeds; (**b**) printed samples of the corresponding LPBF process parameters [52].

The laser energy density can control the crystallization, microcracking, and densification in LPBF-fabricated BMG parts, which determined the mechanical properties of the parts [51,84,111]. During LPBF processing, a reasonable laser energy density can prepare high-density parts. Wang et al. [52] manufactured a Ø 45 × 20 mm Fe_55_Cr_25_Mo_16_B_2_C_2_ block through LPBF and produced BMG with different process parameters. The microstructure after 3D printing remains completely amorphous, achieving a good relative density. At a laser power of 100 W and a scanning speed of 300 mm/s, a nearly fully dense amorphous sample can be achieved, as shown in Figure 18. According to most studies, the reasonable energy density of Fe-based BMG ranges from 30 to 100 J/mm^3^ [50,52,54,84,97,107,108,112]. Hofmann et al. [50] investigated the effect of energy densities ranging from 20 to 300 J/mm^3^ on the microstructure and properties of an FeCrMoBC BMG. In this study, they found that high energy densities resulted in the excessive melting of the BMG and cracking during solidification, while low energy densities resulted in parts that sintered loosely and could not be formed.

Based on the above analysis of the LPBF process parameters, process monitoring and control requirements can be introduced. Through real-time process monitoring, the LPBF process parameters can be adjusted so as to avoid the formation of relevant defects and prepare BMG parts with desired quality. Conceptually, the melt pool, as the smallest manufacturing unit of LPBF, has a direct impact on the quality and performance of the final part in terms of both geometry and stability. In addition, the melt pool morphology is influenced using LPBF process parameters such as laser power and scanning speed. Therefore, the monitoring and control of the electromagnetic characteristics associated with the melt pool has been the subject of most LPBF process monitoring research and development efforts.

However, no research literature related to the process monitoring and control of LPBF-fabricated BMGs has been published to date. It can be predicted that defects can be effectively eliminated by monitoring and controlling the characteristics of the electromagnetic melt pool and setting the process parameters to ideal values during the LPBF procedure.

### 5.2. Modifying the Scanning Strategy

**Figure 19:**
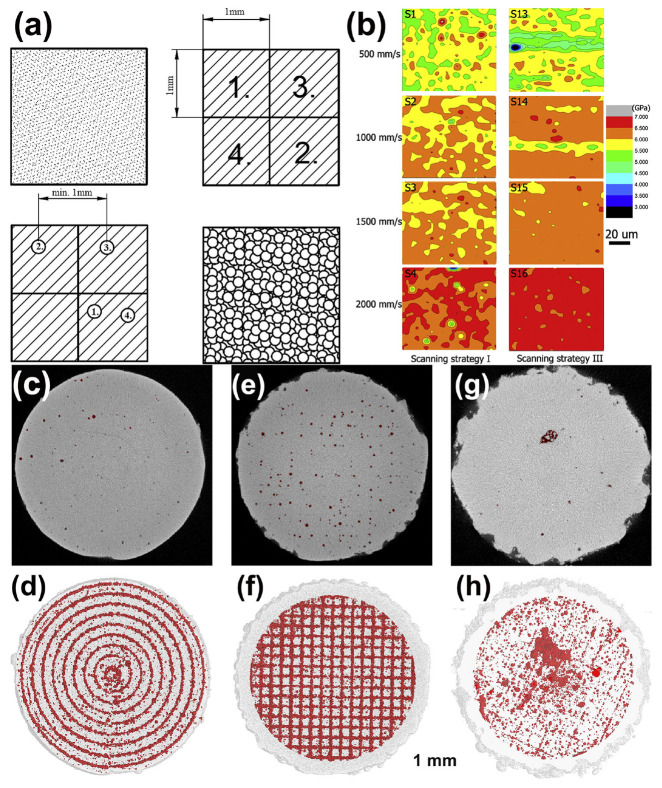
(**a**) P-R scanning strategy: using a chessboard strategy to melt powder for the first time, followed by melting again using random pulses [53]. (**b**) Nanoindentation spectra of Zr_52.5_Ti_5_Cu_17.9_Ni_14.6_Al_10_ BMG under different scanning speeds and strategies [39]. (**c**–**h**) The μ- CT plots and porosity distribution of Zr_52.5_Cu_17.9_Ni_14.6_Al_10_Ti_5_ samples under different scanning strategies. Sample 1: (**c**,**d**), a filling line scan with 200 μm distance (E = 13 J/mm^3^, ρ_Relative_ = 97.7%). Sample 2: (**e**,**f**), a chessboard strategy (E = 12 J/mm^3^ ρ_Relative_ = 97.2%). Sample 3: (**g**,**h**), a unidirectional scanning vector rotated 90 ° in adjacent layers (E = 13 J/mm^3^, ρ_Relative_ = 98.5%) [40].

The quality of BMG formed parts depends not only on the process parameters but also on the scanning strategy. In addition to conventional linear scanning, studies have reported multiple scanning [39], two-step (point-random) scanning strategy (Figure 19a) [53] and layer-by-layer rotational 90° laser scanning modes [35].

Li et al. [39] reported that the use of a multiple scanning strategy successfully overcame the elemental segregation and chemical heterogeneity, which resulted in a more homogeneous elemental distribution and improved the average hardness of Zr-based BMGs, as shown in Figure 19b. The method of using multiple scans to increase the proportion of amorphous phase is applicable to both Fe-based and Zr-based amorphous materials. Subsequently, Li et al. [58] used a remelting method to prevent cracks and growth. A higher power was used to melt the powder in the first scan, and a lower laser power was used in the second scan to release the stress. Although crack-free parts were prepared, this method could easily lead to the severe crystallization of the BMG parts.

Ż Rodowski et al. [53] studied the point random (P-R) strategy, as shown in Figure 19a. The results show that the fraction of amorphous phase can be maximized by laser melting in the first stage and short pulses of laser in the second stage. For the LPBF of an FeSiBCCr alloy, by optimizing the scanning strategy, the amorphous degree value increased from 62.3% to 89.6%.

Zou et al. [108] found that by combining laser remelting and chessboard scanning strategies, the residual stress was reduced to 29% and thermal stress accumulation was reduced. The thermal stress and residual stress of Fe-based MGs were significantly reduced, providing a new approach for the formation of Fe-based BMG components devoid of cracks.

Pauly et al. [40] examined μ-CT pictures of Zr-based BMGs that were created utilizing various scanning strategies (Figure 19c), and found that a scanning strategy with a constant hatch spacing of 200 μm produced the lowest surface roughness, with the pores arranged in a circular pattern. The checkerboard scanning method resulted in LPBF-fabricated BMG components with pores clustered at the centers or edges of each scanning line. In contrast, the unidirectional scanning strategy with 90° rotation between layers had the lowest porosity and showed a random distribution. Therefore, different scanning strategies have significant effects on the porosity and distribution, and the optimization of scanning strategies to increase the densification can also improve the quality of LPBF-fabricated BMG parts.

## 6. Conclusions

In this work, we present a review of the mechanical properties of LPBF additively manufactured BMGs. The BMG alloy systems that have been printed using LPBF have been briefly classified and summarized. The mechanical properties of LPBF-fabricated BMGs including the micro- and nano-hardness, micropillar compressive behavior, and macro-compressive and tensile behavior have been summarized and analyzed. Numerous studies have revealed that the presence of defects in LPBF-fabricated BMGs, including the porosity, LoF, microcracks, crystallization, and HAZ, plays a decisive role in weakening their mechanical properties. The efforts to improve the quality and mechanical performance of LPBF-printed BMGs, such as parameter optimization and scanning strategy modification have been summarized and commented. The strength of LPBF additively manufactured BMGs is significantly lower than that of their cast counterparts due to the existence of defects in the as-built BMG parts. However, their strength can be further enhanced by controlling defects in the as-printed BMGs (reducing or even eliminating defects). It can be foreseen that LPBF is expected to develop into mainstream manufacturing technology for large-sized and complex-shaped BMG components.

## Figures and Tables

**Table 1:** Summary of the BMGs fabricated using LPBF and their structural state.

Composition (at%)	Amorphization Degree (XRD)	Crystallinity (DSC)	Crystalline Phase	Ref.
Zr_52.5_Cu_17.9_Ni_14.6_Al_10_Ti_5_	Amorphous	-	-	[39]
Zr_52.5_Cu_17.9_Ni_14.6_Al_10_Ti_5_	Amorphous	4.1%	-	[40]
Zr_55_Cu_30_Al_10_Ni_5_	Partially crystalline	16.9%	Al_5_Ni_3_Zr_2_	[41,42]
Zr_57.4_Ni_8.2_Cu_16.4_Ta_8_Al_10_	Partially crystalline	8%	Ta	[43]
Zr_59.3_Cu_28.8_Nb_1.5_Al_10.4_	Amorphous	4.0%	-	[44,45]
Zr_60.14_Cu_22.31_Fe_4.85_Al_9.7_Ag_3_	Amorphous	5.3%	-	[46,47]
Zr_60_Fe_10_Cu_20_Al_10_	Amorphous	3.1%	-	[48]
Fe_54.35_Cr_18.47_Mn_2.05_Mo_13.93_W_5.77_B_3.22_C_0.90_Si_1.32_	Amorphous	-	-	[35]
Fe_37.5_Cr_27.5_Mo_10_C_12_B_13_	Amorphous	-	-	[49,50]
Fe_68.3_C_6.9_Si_2.5_B_6.7_P_8.7_Cr_2.3_Mo_2.5_Al_2.1_	Amorphous	2%	-	[51]
Fe_55_Cr_25_Mo_16_B_2_C_2_	Amorphous	0.8%	-	[52]
Fe_71_Si_10_B_11_C_6_Cr_2_	Partially crystalline	10.4%	Fe_2_B, Fe_3_B, α-Fe(Si), Fe_3_Si	[53]
Fe_74_Mo_4_P_10_C_7.5_B_2.5_Si_2_	Partially crystalline	-	α-Fe, γ-Fe, Fe_23_B_6_	[38]
Fe_70_Cr_5_Ni_3_Mo_3_W_9_Si_5_B_5_	Partially crystalline	20%	α-Fe, Fe_2_B	[54]
Fe_43.7_Co_7.3_Cr_14.7_Mo_12.6_C_15.5_B_4.3_Y_1.9_	Partially crystalline	36.1%	(Fe,Cr)_23_(C,B)_6_, (Fe,Cr)_23_B	[55]
Ti_47_Cu_38_Zr_7.5_Fe_2.5_Sn_2_Si_1_Ag_2_	Amorphous	-	-	[56]
Al_86_Ni_6_Y_4.5_Co_2_La_1.5_	Partially crystalline	-	-	[57]
Al_85_Ni_5_Y_6_Co_2_Fe_2_	Fully crystallized	-	-	[58]
Al_85_Nd_8_Ni_5_Co_2_	Partially crystalline	-	α-Al, AlNdNi_4_, Al_4_CoNi_2_, AlNd_3_	[59]
Cu_46_Zr_47_Al_6_Co_1_	Partially crystalline	-	B2-CuZr	[60]
Cu_50_Zr_43_Al_7_	Partially crystalline	18.9%	CuZr, Cu_10_Zr_7_, Al_2_Zr	[61]
Cu_46_Zr_46_Al_8_	Partially crystalline	12%	B2-ZrCu, cube phase	[62]
Cu_50_Zr_50_	Partially crystalline	37.1%	B2-ZrCu, B19′-ZrCu, Cu_5_Zr	[63]
Pd_43_Cu_27_Ni_10_P_20_	Amorphous	-	-	[64]

**Table 2:** Microhardness of BMGs produced via the LPBF technique.

Alloy	Hardness (HV)	Load (kgf)	Ref.
Zr_59.3_Cu_28.8_Nb_1.5_Al_10.4_(AMZ4)	465 455 446	1 2 5	[36]
Zr_59.3_Cu_28.8_Nb_1.5_Al_10.4_(AMZ4)	484 469 466	5 0.5 0.05	[80]
Zr_59.3_Cu_28.8_Nb_1.5_Al_10.4_(AMZ4)	438	2	[81]
Zr_59.3_Cu_28.8_Nb_1.5_Al_10.4_(AMZ4)	466	2	[82]
Zr_60.14_Cu_22.31_Fe_4.85_Al_9.7_Ag_3_	425	0.5	[46]
FeSiBCrC	900	0.1	[83]
Cu_50_Zr_50_	593 462	0.05	[63]
Pd_43_Cu_27_Ni_10_P_20_	498	1	[64]
	539	0.1	

**Table 3:** Summary of reported compression performance test results for BMGs fabricated using the LPBF method.

Alloy	σ_max_ (MPa)	ε_p_ (%)	Size (mm^3^)	Ref.
Zr_52.5_Cu_17.9_Ni_14.6_Al_10_Ti_5_ (Vit105)	1500	0	Ø3 × 6	[39]
Zr_52.5_Cu_17.9_Ni_14.6_Al_10_Ti_5_ (Vit105)	1670	<0.5	Ø3 × 6	[40]
Zr_52.5_Cu_17.9_Ni_14.6_Al_10_Ti_5_ (Vit105)	1710	0.5	Ø3 × 6	[85]
Zr_52.5_Cu_17.9_Ni_14.6_Al_10_Ti_5_ (Vit105)	1710	0.5	Ø3 × 6	[94]
Zr_55_Cu_30_Al_10_N_i5_	1504	0	Ø3 × 6	[41]
Zr_55_Cu_30_Al_10_Ni_5_	1499	0	Ø3 × 6	[89]
Zr_57.4_Ni_8.2_Cu_16.4_Ta_8_Al_10_	1932	2.15	Ø1.5 × 3	[43]
Zr_59.3_Cu_28.8_Nb_1.5_Al_10.4_(AMZ4)	1368	0	Ø6 × 9	[36]
Zr_59.3_Cu_28.8_Nb_1.5_Al_10.4_(AMZ4)	1860	2.03	Ø0.002 × 0.005	[82]
Zr_60.14_Cu_22.31_Fe_4.85_Al_9.7_Ag_3_	1770	0.4	Ø3 × 6	[46]
Zr_60.14_Cu_22.31_Fe_4.85_Al_9.7_Ag_3_	1849	1.04	Ø2 × 4	[95]
Fe_55_Cr_25_Mo_16_B_2_C_2_	6000	<2	Ø0.0015 × 0.003	[52]
Fe_43.7_Co_7.3_C_14.7_B_4.3_Mo_12.6_Cr_15.5_Y_1.9_	100	0	Ø2 × 4	[93]
Ti_47_Cu_38_Zr_7.5_Fe_2.5_Sn_2_Si_1_Ag_2_	1690	0	Ø2 × 4	[56]
Mg_65_Cu_20_Zn_5_Y_10_	467	0	2 ×2 ×4	[96]
Al_85_Nd_8_Ni_5_Co_2_	1080	2.45	N. A	[59]
Cu_46_Zr_47_Al_6_Co_1_	940	0	N. A	[60]
Cu_50_Zr_43_Al_7_	1044	0	Ø4 × 8	[61]
Cu_46_Zr_46_Al_8_	1560	0	Ø3 × 6	[62]
Cu_50_Zr_50_	1841	3.17	Ø2 × 4	[63]
Cu/FeCrMoCB	885	2.77	Ø3 × 6	[97]
Pd_47_Cu_23_Ni_10_P_20_	1138	0	Ø4 × 6	[64]

**Table 4:** Summary of the optimal process parameters for amorphous formation of BMG fabricated using LPBF.

Alloy	Laser Power (W)	Scan Speed (mm/s)	Hatch Spacing (mm)	Thickness (mm)	Energy Density (J/mm^3^)	Ref.
Zr_52.5_Cu_17.9_Ni_14.6_Al_10_Ti_5_	400	2000	0.15	0.1	13.3	[39]
Zr_52.5_Cu_17.9_Ni_14.6_Al_10_Ti_5_	105	1000	0.2	0.04	13.1	[40]
Zr_52.5_Cu_17.9_Ni_14.6_Al_10_Ti_5_	109.5	1000	0.2	0.04	13.7	[85]
Zr_52.5_Cu_17.9_Ni_14.6_Al_10_Ti_5_	109.5	1000	0.2	0.04	13.7	[94]
Zr_55_Cu_30_Al_10_N_i5_	240	1200	0.1	0.06	33.3	[41]
Zr_55_Cu_30_Al_10_Ni_5_	240	1200	0.1	0.06	33.3	[89]
Zr_57.4_Ni_8.2_Cu_16.4_Ta_8_Al_10_	100~300	1200~2000	0.1	0.06	22.9	[43]
Zr_59.3_Cu_28.8_Nb_1.5_Al_10.4_ (AMZ4)	30	600	0.09	0.02	27.8	[36]
Zr_60.14_Cu_22.31_Fe_4.85_Al_9.7_Ag_3_	200	1600	0.1	0.06	20.8	[46]
Zr_60.14_Cu_22.31_Fe_4.85_Al_9.7_Ag_3_	160	1200	0.1	0.06	22.2	[95]
Fe_68.3_C_6.9_Si_2.5_B_6.7_P_8.7_Cr_2.3_Mo_2.5_Al_2.1_	340	1500	0.11	0.075	27.5	[51]
Fe_55_Cr_25_Mo_16_B_2_C_2_	100	300	0.105	0.03	105.8	[52]
Fe_74_Mo_4_P_10_C_7.5_B_2.5_Si_2_	320	3470	0.124	0.05	14.9	[38]
Ti_47_Cu_38_Zr_7.5_Fe_2.5_Sn_2_Si_1_Ag_2_	60	2000	0.14	0.04	5.4	[56]
Mg_65_Cu_20_Zn_5_Y_10_	100	1000	0.06	0.05	33.3	[96]
Al_86_Ni_6_Y_4.5_Co_2_La_1.5_	120	1000	-	-	-	[57]
Al_85_Ni_5_Y_6_Co_2_Fe_2_	200 (80)	625	0.15	0.05	42.7 (17.1)	[58]
Al_85_Nd_8_Ni_5_Co_2_	320	1455	0.11	0.05	40.0	[59]
Cu_50_Zr_43_Al_7_	150	2000	0.1	0.03	25	[61]
Cu_46_Zr_46_Al_8_	99	1000	0.18	0.04	14.3	[62]
Pd_47_Cu_23_Ni_10_P_20_	60	600	0.15	0.04	4.7	[64]

## Data Availability

Not applicable.
